# Notes on Miscellaneous Subjects Contained in Foreign and Home Medical Journals

**Published:** 1850-07

**Authors:** Theodore S. Bell

**Affiliations:** Louisville, Kentucky


Art. III.—*Notes on Miscellaneous Subjects Contained in Foreign and Home Medical Journals.* By Theodore S. Bell, M. D., Louisville, Kentucky.

The *Medical Examiner*, published in Philadelphia, Pennsylvania, for June, contains a paper from the pen of Samuel Jackson, M. D., President of the Philadelphia

County Medical Society, entitled "Some Hints on the Comparative Influence of Dry and Moist Air in the Causation of Disease." The drift of the essay is an attempt to show that the sprinkling of streets in cities, with water, is deleterious to health. In a hygienic point of view, the question, started by Dr. Jackson, is one of some importance, and for this reason we proceed to examine it. It seems to us, that Dr. Jackson set about his investigation with a confused set of ideas on the subject, and that the confusion increased greatly towards the close of his paper. Where a number of causes of disease are in operation, it is difficult to select a particular one and mark with precision the boundaries of its influence. An idea was once prevalent, that fogs were the cause of sickness in many portions of the West and South. Dr. Wistar noticed that certain laborers on his place were affected with fever, while others were free from it. He observed that heavy fogs, passed up certain ravines and gullies, to the residences of the sickly set, while the healthy ones were not subject to such a visitation. The inference that the humid atmosphere was the cause of fever seemed accurate, both from the negative and affirmative fact. This error arose, however, from precisely the same kind of reasoning that Dr. Jackson makes in his paper; a reasoning that presses a solitary item beyond its powers, and neglects essential features of investigation. In the case referred to, on Dr. Wistar's place, the malaria carried in the fog was lost sight of; and a similar neglect caused a wide-spread error to occupy the South and West for a long time. That fogs alone were not the cause of sickness was indubitably established by the fact that the fishermen of New Foundland live for weeks in the heaviest fogs, without any deleterious results. And this fact should have some weight with Dr. Jackson.

That the sprinkling of water, on streets paved with stone cannot have a baleful influence is manifest from a great variety of facts. Before the streets of Louisville were paved, the rains of summer caused a great mortality. This was not on account of the mere humidity of the atmosphere, for the rains that fall in summer now, make the air as humid as that of 1822, but without any evil effect. All parts of the city of Louisville are equally sprinkled by rains, but all parts are not alike unhealthy. A considerable portion of the city retains its health, amidst the heats of summer, the blasts of autumn, the sleets of winter, and the rains of spring, and it is, therefore, evident, that we must look for something else besides the mere dryness or humidity of the atmosphere, as a cause of disease. The drenching rains of summer that fell for a month, in Lexington, Kentucky, in 1833, and caused a frightful cholera epidemic, did not affect all parts of that city alike. The ravages of the epidemic radiated from an unpaved portion of the city, but large squares in Lexington were exempt from the epidemic disease. But the exempted, equally with the afflicted portions had a humid atmosphere. That portion of the city of Cincinnati that is most constantly sprinkled with water from the hydrants is very far from being the most sickly part of the city. And that portion of Louisville most constantly sprinkled by water-carts is precisely the most salubrious part.

But let us survey some other parts of the earth. One of the healthiest portions of Devonshire, in England, is Torquay. It is a resort for a considerable number of invalids. But the humid state of the atmosphere of Torquay is notorious.

The south of Italy has long been celebrated for its salubrity, but it is equally famous for its humid air, and Rome, the most humid part of the south of Italy, has the best climate for invalids. Madeira has long been the shrine of Hygeia, to which pilgrims from all parts of the world have resorted, but it, too, has an atmosphere that can scarcely be called dry.

In the West Indies the hot, dry months are not healthy. The invalid is urged to spend all the time he can upon the water, where the atmosphere is much more humid than that of the land. A ship may be "dry," but its atmosphere is not.

An important fact should also be borne in mind, and that is, that there are many diseases which are benefitted by humid air, while there are others injured by it. While, therefore, Dr. Jackson's hydrophobia might benefit one set of invalids, it would be deleterious to another.

It is utterly impossible that arid streets, and an atmosphere loaded with impalpable dust of carbonate of lime can be salubrious. The experience and observation of all men teach that they are prejudicial to health. And it is well known, we think, that Dr. Jackson's recipe of a well handled broom, in place of the sprinkling of water, would be much more likely to make these causes of diseases, active, rather than dormant.

We have remarked that Dr. Jackson's confusion increases towards the conclusion of his essay. He finds it difficult to meet the objection urged, against his views, in the appeal to the genial influence of showers of rain. He solves the difficulty by declaring that there is no kind of comparison between "pure water from the higher regions, which in its descent, washes and purifies the air," and "the impure and pestiferous water of the Schuylkill." This then is a surrender of the whole question. If Dr. Jackson had started out with the proposition that sprinkling streets with impure and pestiferous water is injurious to health, he would have found no one to controvert his position. Truth forces him to admit that streets may be sprinkled with pure water, without any injurious effects.

But there is another important admission in Dr. Jackson's effort to meet the fact we have mentioned, an admission that is fatal to his entire series of speculations. In announcing that falling rain washes out impurities from the atmosphere, he admits that a dry air may be unwholesome. These impurities spring from the bosom of the earth, and nine-tenths of them are of a character that may be kept from a breathing atmosphere, by sprinkling the streets with pure water. The hydrants and fountains of New York and Boston, are not detrimental to health.

Dr. Jackson closes his hydrophobic essay, in the following words: "The washing of the pavements is a very great evil whether in winter or in summer, independently of the dampness it adds to the atmosphere. If done in the morning, it annoys passengers by wetting their feet, and not unfrequently the pavements become thereby covered with ice and dangerous to all pedestrians; if done in the evening, as it often is, it is sure to have this effect in freezing weather; and in the milder, they remain wet till the next day noon. Now it is a fact, that there are many sick people who cannot take fresh air except by walking, and to them, and indeed to all delicate people, the damp pavements present a very formidable obstacle. This is not an hypothesis of mine, but a stubborn fact from the lips and hearts of many sickly people."

Now this sounds to us very much like what some people call "murmuring at Providence." He who orders all things, has ordained rains for a Philadelphia winter, and without any regard to "sick people who cannot take fresh air, except by walking," or to all delicate people, or to Dr. Jackson's opinion that it is a great evil, Providence has ordained freezing weather for Philadelphia, and we cannot see how Dr. Jackson's strong objections to rain and frozen pavements can alter the movements of Heaven. His remarks apply as forcibly against winter rains as against the use of Schuylkill water. According to Dr. Jackson's hypothesis it is wrong to send rain on a winter's evening, because if the weather is cold the pavements will freeze, and if not cold enough for that, "they will remain wet until next day noon." Dr. Jackson evidently thinks there should be no rain in freezing weather; and that all winter rains should fall in the morning, and Providence should send out a drying sun, to dry up the pavements sooner than next day at noon. Really we cannot see how Dr. Jackson is to be accommodated. Even if the city authorities of Philadelphia could be persuaded to bring water "from the Lehigh, or the Delaware far above the falls," the evil would not be removed, for Providence would prove to be rather an intractable subject for reform on the puny reasonings of an erring mortal. We doubt whether Dr. Jackson has made much of a contribution to Hygienic science.

### NITRATE OF SILVER IN THE CURE OF INFLAMMATION.

In a recent number of this Journal, we gave an abstract of Mr. Higginbottom's paper on the use of nitrate of silver in wounds of the face, lacerated perineum, &c. We have had so much professional experience of its good effects, that we take pleasure in presenting another abstract of Mr. Higginbottom's views, contained in the June number of the *London Lancet.*

Mr. Higginbottom endeavors to remove the idea that nitrate of silver is a caustic in any sense of the word. He says: "It subdues inflammation, and induces resolution, and the healing process. It preserves, and does not destroy, the part to which it is applied. If we compare a caustic, as the hydrate of potassa with the nitrate of silver, we find that the hydrate of potassa destroys and induces a slough, and the ulcerative process; but if we touch a part with the nitrate of silver, the eschar remains for a time and then falls off, leaving the subsequent parts healed."

"If an ulcerated surface, secreting pus, be touched by the nitrate of silver, the succeeding discharge is immediately converted into lymph; it is the property of the hydrate of potassa, on the contrary, to induce not only ulceration but suppuration. In short, the peculiar properties of the nitrate of silver have long been kept unknown to us by the designation of lunar caustic, affording the most striking instance of the influence of a term or of a classification, upon the human mind. The nitrate of silver and the hydrate of potassa (as indeed all caustics) are as the poles to each other; the first preserves, the second destroys; the first induces cicatrization, the second ulceration."

Mr. Higginbottom prefers the nitrate of silver in a concentrated solution, to the solid stick or crystal. The solution is made by dissolving ℈iv nitrate of silver, in ʒiv distilled water. If rightly prepared the solution will be clear and transparent. This solution may be applied with a small piece of sponge, stitched on the eye of a common silver probe. The sponge should be *well* washed after using it.

It is necessary that the surface of the skin, to which the nitrate is to be applied, shall be free from any oleaginous matter, loose cuticle, or any other extraneous substance; the parts should be well washed with soap and water, and afterwards with water alone, to remove any particle of soap remaining."

The precise effect of the nitrate of silver, in different degrees of its application should be clearly understood. "If the nitrate of silver be once passed slightly over the moist skin of any part, except the hands or soles of the feet, (upon which the cuticle is thicker than elsewhere,) it induces an eschar, simply; if it be passed over the surface twice or thrice, to the eschar will be added some vesication; if more still, there will be vesication only. In the first place there will be no pain, in the second and last, there will be soreness proportionate to the degree of vesication. It is essential to this plan of treatment, that these observations be kept constantly in view."

In *recent bruised wounds of the skin, small ulcers, large ulcers, old ulcers of the legs, ulcers attended with inflammation, punctured wounds, bites, and stings, wounds received during dissection, wounds from the bites of rabid animals, lacerated wounds, hemorrhage from leech bites, incised wounds, erysipelas, phlegmonous erysipelas, inflammation of the absorbents, phlegmonous inflammation, small irritable ulcers with varicose veins, burns or scalds, gangrena senilis,* Mr. Higginbottom recommends the use of the nitrate of silver, conjoined with constitutional treatment, diet, rest, &c.

### CHOLERA STATISTICS.

William J. Cox, surgeon, makes a tabular report in the June number of the *London Lancet*, on the treatment of Cholera. The following is the analysis and classification of Mr. Cox's table:

| | | Result. |
|---|---|---|
| Cases treated by stimulants and opium........ | 17.... | 12 deaths. |
| " " " " alone........... | 5.... | 3 " |
| " " " calomel in large doses....... | 10.... | 1 " |
| " " " tartar emetic............... | 4.... | 3 " |
| " " " on Dr. Ayre's system, modified by co-administration of ice, sulphuric acid, and sponging, with aqua regia water................ | 54.... | 13 " |

### CHARCOAL IN NERVOUS-GASTRO-INTESTINAL AFFECTIONS, EITHER OF AN IDIOPATHIC OR SYMPATHETIC KIND.

Dr. Belloc, surgeon of the sixth hussars (France) highly recommends the use of charcoal in the various forms of indigestion, and constipation. We have often seen it very serviceable in such cases.

### COD-LIVER OIL IN TERTIARY SYMPTOMS.

Dr. Manas, of Madrid, commends the use of cod-liver oil in tertiary syphilitic affections. He has found it successful in cases that had resisted the influence of mercury.

### TREATMENT OF CHOREA.

M. Faivre d' Esnans urges the use of Prussiate of iron in chorea. The following is his formula: Prussiate of iron fifteen grains, extract of valerian forty-five grains, made into twenty-four pills. One pill to be taken three times a day, at six hours' interval, each pill to be followed by a wine-glass full of infusion of valerian.

### ACQUIRED IDIOSYNCRACY.

Dr. Sherriff, of Canada, details in the *British American Medical Journal* some curious particulars concerning himself. After an attack of measles, in 1840, he was frequently subject to asthma, and he ascertained by experiment, that these attacks were caused by ipecacuanha. That medicine produces in Dr. Sherriff violent irritation of the mucous membrane of the nose, mouth, and throat. Urgent dyspnea is produced when he takes ipecacuanha. Yellow colored water, soon changed to bloody serum, passes from his nostrils, and the mouth and throat are much inflamed. Prior to Dr. Sherriff's attack of measles he was in the habit of taking ipecacuanha, and of preparing it pharmaceutically, without injury or inconvenience. The idiosyncrasy that now rules Dr. S., has been acquired by measles.

### THE TREATMENT OF EPILEPSY.

The *Journal of Insanity* gives an abstract of Dr. Cheneau's paper, presented to the Academy of Sciences, Paris, on the treatment of epilepsy. Dr. C. believes that the disease is curable by medication, and relies principally on the judicious employment of digitalis. The most inveterate cases have yielded to the persevering use of digitalis for a period of six or eight months. Six instances have been submitted to the academy, in which Dr. Cheneau has been successful.

M. Delasiauve, one of the physicians to the Bicetre, is investigating the resources of medical art in the cure of epilepsy. The following is the result of his investigations thus far:

"To valerian he gives the first place, though he does not speak of it quite in as sanguine language as did Tissot; a decoction of valerian given in doses of two wine glasses full, morning and evening, has produced a radical cure, but the medicine requires to be persevered in for a considerable length of time, otherwise it is of little avail; assafetida decidedly moderates the violence of the access, but it does not seem to produce the same permanent good effect. The hydrocyanate of iron is found in some instances very beneficial; belladonna and digitalis are each serviceable, but Pluvrey of Lille prefers a combination of the two. The root of artemisia is occasionally useful; of liquid ammonia, according to the formula prepared by Martinet, he has some good reason to speak, and will shortly give the results of his experience; camphor is found in those cases where the reproductive system is in a high state of excitement, as not unusually occurs in epileptics, to be of remarkable service; zinc, musk, castor, ambergris, have but little curative power; preparations of copper are to be but little confided in; nitrate of silver has lost the high character it once obtained; sulphate of quinine has also fallen into disrepute. He enters very minutely into the subject of the diet and exercise of the patient, preferring vegetable to animal food. It is to be regretted that the series of papers which this observant practitioner had prepared for the press are for the present suspended, for want of that encouragement which would have been at another period given to inquiries of such deep moment."

CROUP TREATED BY TOPICAL APPLICATION OF NITRATE OF SILVER.

The Buffalo *Medical Journal*, for June, contains a report by Dr. P. H. Strong, of a case of croup treated by the topical application of the nitrate of silver, according to the method presented in Dr. Green's book on croup. The case reported by Dr. Strong is exceedingly interesting. The croup occurred in a child three and a half years of age, and was treated actively with ipecacuanha and tartar emetic, followed by small portions of calomel, and turpeth mineral. The case grew worse, however, and the topical treatment by nitrate of silver was determined on. A solution of the nitrate, ℈ij in ℥i of water was tried, but as the patient was not relieved, the strength of the solution was doubled. Two applications of this latter, seemed to rescue the patient from the very verge of death.

The case thus reported by Dr. Strong is confirmatory of the practical views of Dr. Green, and we are confident that the use of nitrate of silver, beyond what generally obtains with it, will increase the success of medical men in the treatment of disease. We have occupied a good deal of space in this number, with commendations of the application of nitrate of silver in the treatment of inflammations, but not more than it deserves. There are few things among the remedial agents in the hands of physicians that are as worthy of confidence, as the nitrate of silver, and we strongly commend it to the practising physician.

